# Quality and Microbiome Analysis of Pickled Swimming Crabs (*Portunus trituberculatus*) during Storage at Two Alternative Temperatures

**DOI:** 10.3390/molecules28237744

**Published:** 2023-11-24

**Authors:** Yu Chen, Peipei Li, Dan Xu, Xiaojun Zhang, Tao Huang

**Affiliations:** 1 Zhejiang Marine Fisheries Research Institute, Zhoushan 316021, China; chenxiaoyu2141@163.com (Y.C.); liwanzhao999@163.com (P.L.); xdplmm@126.com (D.X.); xiaojun3627@163.com (X.Z.); 2 Scientific Observing and Experimental Station of Fishery Resources for Key Fishing Grounds, Ministry of Agriculture and Rural Affairs, Zhoushan 316021, China; 3 Key Laboratory of Sustainable Utilization of Technology Research for Fishery Resource of Zhejiang Province, Zhoushan 316021, China; 4 State Key Laboratory of Food Science and Technology, Nanchang University, Nanchang 330047, China

**Keywords:** pickled swimming crab, quality, biogenic amine, microbiome analysis, food safety

## Abstract

The storage quality and microbiome analysis of pickled swimming crabs (*Portunus trituberculatus*) stored at 20 and 4 °C were investigated. It showed that samples stored at 4 °C had a longer shelf life, lower total viable count (TVC), pH, and total volatile base nitrogen (TVB-N) contents than those stored at 20 °C. The biogenic amine (BA) results demonstrated that tyramine (tyr), putrescine (put), and cadaverine (cad) were the dominant amines in all samples, and samples stored at 4 °C had lower BA contents. A microbiome analysis indicated that a salt–alcohol water mixture significantly inhibited the growth of *Tenericutes*. *Firmicutes*, *Proteobacteria*, *Bacteroidetes*, *Acidobacteria*, *Actinobacteria*, and *Cyanobacteria* were the dominant bacteria of stored pickled crabs, and storage at 4 °C significantly inhibited the growth of dominant bacteria, more than that of 20 °C. In conclusion, 4 °C storage guaranteed the quality of samples by inhibiting changes in biochemical properties and the growth of dominant bacteria, thereby prolonging its shelf life.

## 1. Introduction

The swimming crab (*Portunus trituberculatus*) is a highly rated seawater crustacean native to East China and other East Asian countries. In China, swimming crabs are a major aquacultural fish species, with approximately 5.3 million tons produced in 2020 [1]. The swimming crab is popular for its delicious, digestible, highly nutritious meat, which is rich in DHA, EPA, vitamins, and minerals [2]. The crab is mainly sold live, and meat from the body and legs is processed into cooked–frozen meat [3] or instant food. Pickled swimming crab is a popular delicate instant food product in the coastal areas of Zhejiang, China; it is easily obtained by sousing swimming crab in salt and white wine in refrigerated conditions for 12 h.

Crustaceans are highly prone to spoilage during storage due to their large quantities of free amino acids and nitrogenous compounds, which reduce freshness and shelf life [4,5]. Spoilage is mainly associated with microbial activity, which can be evaluated through a combination of measurements, including microbial growth as well as sensory, biochemical, and physical qualities [5]. Low temperature preservation is a demonstrably important preservation method for fishery products because it minimizes undesirable chemical reactions and slows microbial growth [2,6,7]. The pickled swimming crab is domestically stored at 4 or 20 °C, although this is not a good manufacturing practice, according to the guidelines of the current Codex Alimentarius [8]; however, it is still widely practiced by domestic consumers in the Zhejiang coastal region. However, there is still a lack of basic information on pickled swimming crab, especially in terms of the serious quality and safety concerns for consumers.

Thus, the aim of this study was to evaluate the quality of pickled swimming crab during family storage conditions (4 and 20 °C) through analysis of sensory scores and biochemical properties (TVB-N, TVC, pH, and BAs). Furthermore, the dominant microorganisms were also evaluated using 16S rRNA gene high-throughput sequencing. This study was anticipated to provide useful information to help consumers understand pickled crab characteristics.

## 2. Results and Discussion

### 2.1. Sensory Analysis

**Figure 1:**
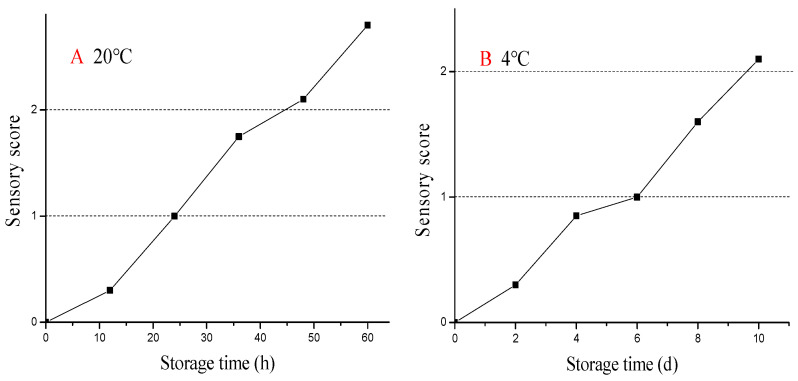
Storage temperature’s effect on the sensory evaluation (**A**,**B**) of pickled crabs during storage.

Sensory evaluation is a technique used to analyze quality changes in pickled crab, and it is generally highly correlated with other quality indices. As shown in Figure 1A,B, crab stored at 4 °C had higher sensory scores than samples stored at 20 °C, indicating that low-temperature storage extends the shelf life of pickled swimming crab.

In high-temperature storage (20 °C), the initial quality characteristics changed from a bright appearance, firm meat, and muscles strongly attached to the shell to a dark exterior and loose meat, leading to HQL being reached after storage for 24 h, and sensory scores over two (SL) after 48 h of storage. This might be due to the enzymatic activity from the hepatopancreas and mirobial breeding [8]. At low temperature (4 °C), the samples had highly acceptable sensory characteristics during long-term storage (6 days), but decomposition was observed with the formation of a bad smell and loose tissue, causing unacceptable scores after 10 days. In this study, low storage temperature (4 °C) led to high sensory evaluation, indicating that low-temperature storage could extend the shelf life of pickled swimming crab and guarantee its quality.

### 2.2. Microbiological Analysis

**Figure 2:**
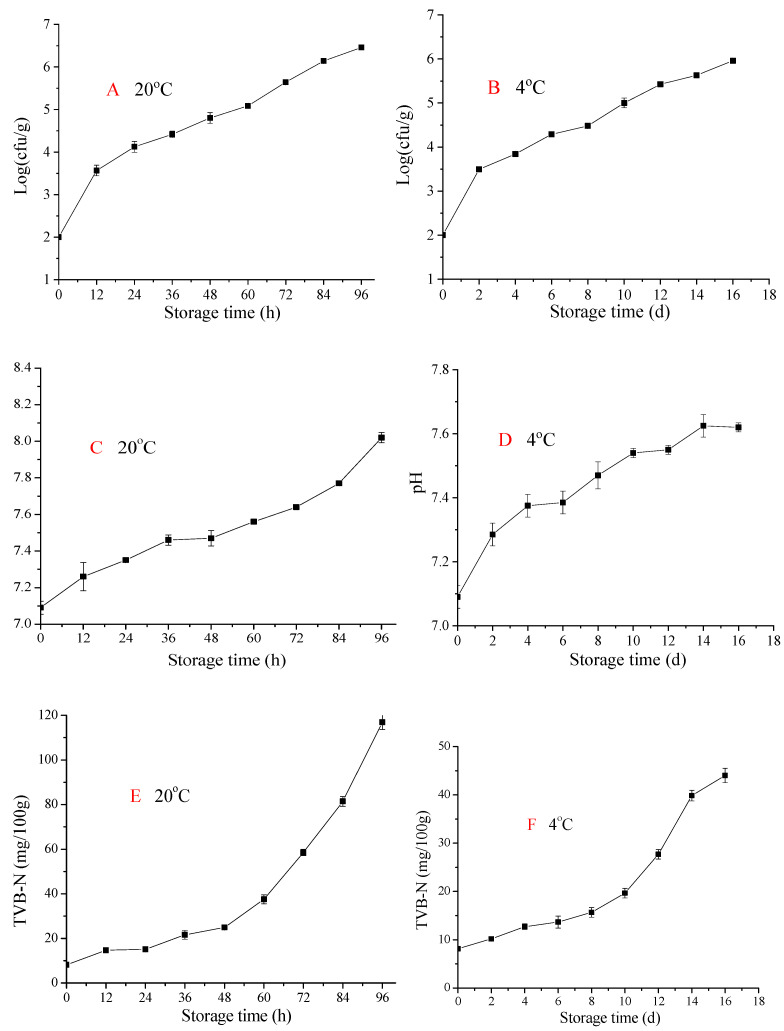
Effect of storage temperature on the TVC (**A**,**B**), pH (**C**,**D**) and TVB-N (**E**,**F**) of pickled crabs during storage.

Microbial activity is one of the major factors responsible for seafood spoilage; microbiological analysis can help to elucidate quality and safety properties during storage [9]. Changes in bacterial enumerations log (cfu/g) of pickled crabs stored at 20 and 4 °C are shown in Figure 2. The initial TVC of pickled crab was 1.99 ± 0.02 log (cfu/g), which was slightly higher than that of raw swimming crab 2.48 ± 0.13 log (cfu/g), raw snow crab 2.50 log (cfu/g), and raw blue crab 2.50 log (cfu/g). This might be because the alcohol and salt inhibited bacterial growth during pickled swimming crab preparation.

In samples stored at 20 °C (Figure 2A), TVC significantly increased from 1.99 ± 0.02 to 5.08 ± 0.02 log (cfu/g) as storage time increased from 0 to 60 h; the increase in TVC slowed after 60 h. Meanwhile, for samples stored at 4 °C, TVC slowly increased from 1.99 ± 0.02 to 3.84 ± 0.04 log (cfu/g) when stored for 4 days (>96 h). These results demonstrate that low-temperature storage can effectively inhibit microbial growth. According to the International Commission on Microbiological Specifications for Foods and Chinese regulations [10], the acceptable TVC limit for marinated seafood is 5 log (cfu/g), and the end of shelf life for samples stored at 20 and 4 °C should be 70 h and 16 days, respectively; thus, low-temperature storage can prolong the shelf life of pickled crab. This is similar to Hernández et al. [9], who reported that low-temperature storage (4 °C) could prolong shelf life of aquacultured meagre (*Argyrosomus regius*) fillet. Moreover, in this study, microbiological analysis results correlated well with sensory scores.

### 2.3. pH Analysis

pH can be used as an indicator of the extent of proteolysis. As shown in Figure 2C,D, pH sharply increased from 7.09 ± 0.04 to 8.02 ± 0.03 and 7.62 ± 0.01 for samples stored at 20 °C and 4 °C, respectively. This might be due to protein decomposition and deamination by microbes and enzymes [6]. Samples stored at 4 °C had lower pH values than those stored at 20 °C, indicating that low-temperature storage (4 °C) strongly inhibited proteolysis compared to 20 °C. Moreover, the pH of meat products mainly depends on the free carboxyl and amino groups in low-molecular-weight compounds and cellular macromolecules (e.g., proteins, nucleic acids, and polysaccharides) [6].

### 2.4. TVB-N Evaluation

TVB-N can be used as an indicator of a food’s fitness for consumption [11]. TVB-N of crab is mainly related to sex, season, harvesting area, age, and species [12]. According to the Chinese regulations [10], the acceptable TVB-N limit for marinated seafood is 25 mg/100 g. As shown in Figure 2E,F, TVB-N values increased during storage at different temperatures (*p* < 0.05). Briefly, the initial TVB-N value was approximately 8.16 ± 0.47 mg/100 g. At 20 °C, TVB-N significantly increased to 24.94 ± 0.11 mg/100 g at 48 h, which is close to the unacceptable limit (25 mg/100 g), and then increased to 37.48 ± 0.95 mg/100 g after 60 h. Meanwhile, TVB-N increased slowly over 8 days of storage at 4 °C, reaching 19.67 ± 0.58 mg/100 g after 10 days *(p* < 0.05). Similarly, Xu, Xia, and Kim [13] reported that the TVB-N of Chinese mitten crab increased from 9.87 to 17.87 mg/100 g during 72 h of storage at 4 °C. Parlapani et al. [10] found that the TVB-N of blue crab also increased during storage, and reached higher values at higher storage temperatures. During storage, the increase in TVB-N might be due to nucleotide autolysis and the formation of nitrogenous compounds, as well as bacterial enzymes’ action on trimethylamine oxide [13]. Furthermore, bacterial growth is a very important factor in increasing TVB-N by breaking down proteins and forming volatile nitrogen compounds, thereby producing an unappealing odor and flavor [14,15]. Therefore, compared with at 20 °C, storage at 4 °C significantly decreased TVB-N. This may be due to inhibited bacterial growth in the crab meat.

### 2.5. BAs Analysis

**Figure 3:**
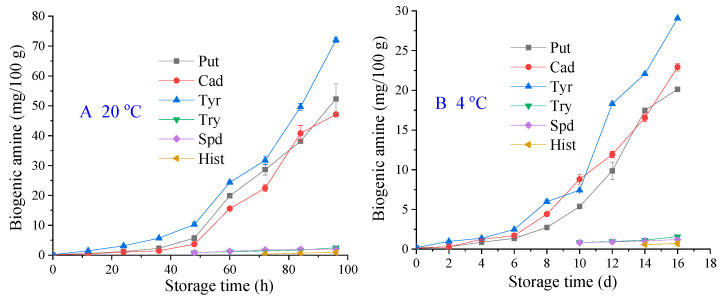
Changes in the concentration of biogenic amines (mg/100 g) in pickled crabs stored at 20 °C (**A**) and 4 °C (**B**).

The average BA values during storage at 20 and 4 °C are presented in Figure 3. Six BAs were investigated: tyramine (tyr), putrescine (put), cadaverine (cad), tryptamine (try), spermidine (spd), and histamine (hist). For the samples stored at 20 and 4 °C, tyr, put, and cad were detected in the early stages, after which they increased sharply and became the dominant amines. Cad and put are two important spoilage indicators in aquatic products because of their ability to potentiate histamine toxicity [16]. For samples stored at 20 °C, tyr, put, and cad slightly increased with storage time from 0 to 36 h, after which they increased sharply; tyr showed the highest increasing rate. After 36 h, tyr, put, and cad levels were 5.72 ± 0.09, 1.48 ± 0.01, and 2.30 ± 0.06, respectively; at the end of the storage period (96 h), tyr, put, and cad levels were 72.02 ± 0.80, 47.14 ± 0.69, and 52.28 ± 5.08, respectively. For the samples stored at 4 °C, after 8 d of storage, the tyr, put, and cad levels were 5.97 ± 0.21, 4.40 ± 0.17, and 2.73 ± 0.03, respectively, but at the end of the storage period (16 d), these were 29.07 ± 0.07, 22.92 ± 0.49, and 20.14 ± 0.13, respectively. These results demonstrate that low-temperature storage can inhibit increasing levels of tyr, put, and cad.

Compared with other three BAs, all samples had lower try, spd, and hist contents, which were <3.0. In fact, neither try, spd, nor hist could be detected at first. Try and spd were first detected at 48 h and 10 d of storage at 20 and 4 °C, respectively. Hist was detected at 72 h and 14 d of storage at 20 and 4 °C, respectively. Among the BAs evaluated, histamine is of particular concern because of its toxicity and allergenic properties [8]. Compared to 20 °C, storage at 4 °C significantly decreased hist content, which is similar to the results of Noori, Khanzadi, Fazlara, Najafzadehvarzi, and Azizzadeh [16], who found less hist in refrigerated common carp during storage. Spd occurs naturally in food, and its formation is not associated with bacterial spoilage [8,17,18]. The present study showed that spd levels increased during storage. This is similar to the findings of Anupama, Laly, Kumar, Sankar, and Ninan [8], who reported that the spd content in crucifix crab body meat increased with storage time at 4 °C. Furthermore, at 20 °C, the total BAs reached 128.52 mg/100 g by 84 h, which was much higher than that of samples stored at 4 °C (75.59 mg/100 g); this also exceeded the BA concentration limit set by the FDA (100 mg/100 g) [19]. Therefore, in combination with sensory score, TVC, pH, TVB-N, and BA trends, low temperature helped to maintain the stable quality of pickled crab.

### 2.6. Composition of Bacterial Community

**Figure 4:**
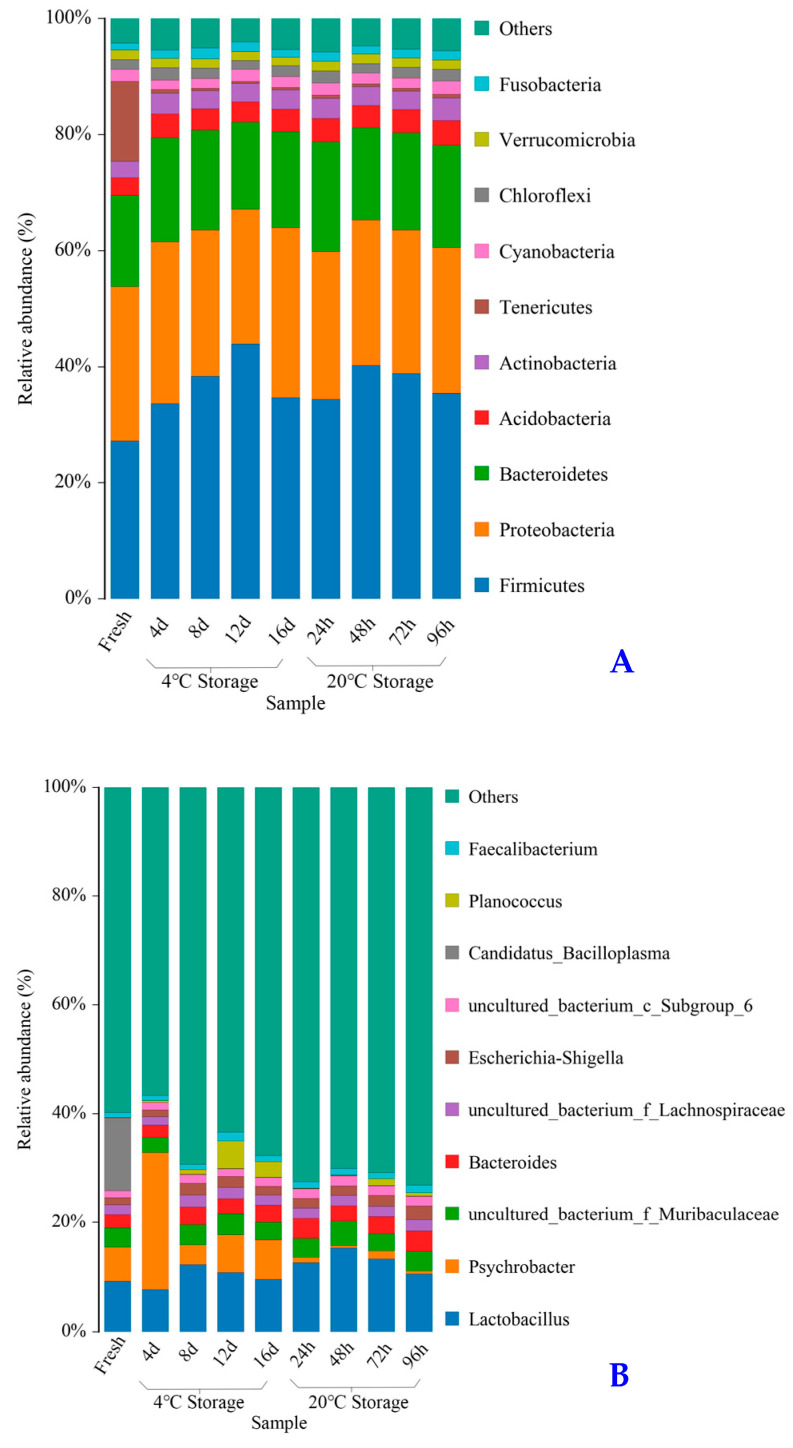
Comparison of bacteria groups in the samples at phylum level (**A**) and at genus level (**B**). “d”: storage day.

Based on the sequencing results, 23,050 operational taxonomic units (OTUs) of all samples were generated under 97% similarity. The coverage was ≥95%, meaning most microbial phylotypes were detected. OTUs of the same kind were analyzed at the phylum and genus levels, and the distributions of dominant bacteria are shown in Figure 4. At the phylum level, the dominant bacteria with relative abundance > 2% in the fresh crab were *Firmicutes* (27.19%), *Proteobacteria* (26.63%), *Bacteroidetes* (15.73%), *Tenericutes* (13.71%), *Acidobacteria* (3.02%), *Actinobacteria* (2.86%), and *Cyanobacteria* (2.11%) (Table 1). In the crab samples stored at 4 and 20 °C, the dominant bacteria were *Firmicutes, Proteobacteria*, *Bacteroidetes*, *Acidobacteria*, *Actinobacteria*, and *Cyanobacteria*. This indicates that a salt–alcohol water mixture significantly inhibited *Tenericutes* growth. Compared with fresh crab, the relative abundance values of all the dominant bacteria of stored crabs slightly increased during storage, especially *Firmicutes*, *Proteobacteria*, *Bacteroidetes*, *Acidobacteria*, and *Actinobacteria*. *Firmicutes* (Gram-positive bacteria) and *Proteobacteria* (Gram-negative bacteria) can negatively affect the safety and quality of food [20]. *Actinobacterias*, a probiotic in humans and animals, was significantly higher in pickled swimming crabs than in fresh crabs [21]. Moreover, the growth rates of the dominant bacteria at 4 °C were slower than those at 20 °C. This indicates that low-temperature storage can inhibit the growth of dominant bacteria and prolong the shelf life of pickled crab.

At the genus level, the initial dominant bacteria (relative abundance > 2%) in the fresh crab were *Candidatus_Bacilloplasma* (13.41%), *Lactobacillus* (9.21%), *Psychrobacter* (6.26%), *uncultured_bacterium_f_Muribaculaceae* (3.45%), and *Bacteroides* (2.52%). In the samples stored at 4 °C, *Lactobacillus, Psychrobacter*, *Bacteroides*, and *Escherichia-Shigella* were the predominant bacteria after 8 days of storage. Meanwhile, in the samples stored at 20 °C, *Lactobacillus*, *uncultured_bacterium_f_Muribaculaceae*, and *Bacteroides* were the predominant bacteria after 48 h of storage. During storage, *Lactobacillus* accounted for the highest proportion of bacteria, and it first increased then decreased for pickled crab samples stored at both 4 and 20 °C. *Lactobacillus* is an essential lactic acid bacteria strain used in traditional fermentation, and it has been found previously in marine products [20]. Interestingly, for samples stored at 4 °C, *Psychrobacter* slightly decreased from 25.10 to 3.58% from 4 to 8 d, and then increased from 3.58 to 7.24% from 8 to 16 d. *Psychrobacter*, a novel marine probiotic, played an important role in the degradation of organics based on unique lipase activity. *Psychrobacter* sp. also exhibited strong potential for producing aroma compounds with pronounced “cheese” notes, and improving the flavor qualities of cheese [22]. The high abundance of *Psychrobacter* significantly increased the total soluble nitrogen content and amino acid nitrogen content, and promoted the umami taste and meaty aroma of crab paste [23]. *Candidatus_Bacilloplasma*, which belongs to the phylum Tenericutes, was exclusively dominant in fresh samples. Some previous studies have reported that the *Candidatus_Bacilloplasma* exhibited is normally present in healthy crustaceans [23,24], and it exhibited a significant decrease in abundance in WSSV-infected shrimps [25]. In the present study, *Candidatus_Bacilloplasma* was not detected in crabs stored at either temperature. This indicates that a salt–alcohol water mixture can significantly inhibit *Candidatus_Bacilloplasma* growth, which is consistent with the finding of Liu [26].

### 2.7. Bacterial Diversity and Difference

Species richness was assessed using the Chao and ACE indices (Table 2). Among all the samples, fresh swimming crabs had the highest ACE (2282.473) and Chao1 (2285.9071) indices. During the early stage of 4 °C-treated sample, both ACE and Chao1 decreased gradually, followed by a rebound in the later stage of storage, which was similar to the trend in the 20 °C treatments. The bacterial community diversity of fresh swimming crabs was the lowest for both indices. These results indicated that crabs with salt and alcohol added had much more complex bacterial communities. Furthermore, the mean coverage of each group was greater than 99.7% (range of 99.73–99.82%), suggesting that the sequencing depth adequately represented the bacterial community.

**Figure 5:**
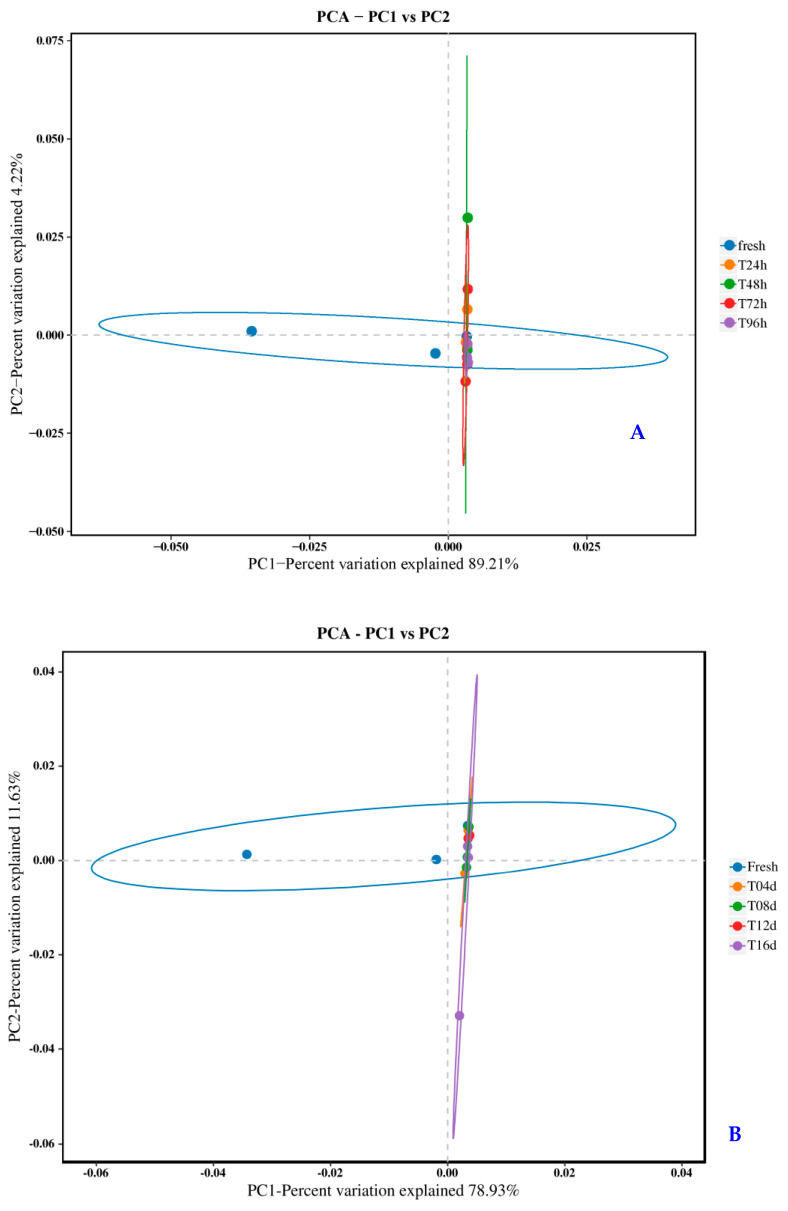
Structural differences in the bacterial communities of pickled crabs stored at 20 °C (**A**) and 4 °C (**B**).

Beta diversity analysis was used to compare the degree of similarity in species diversity among the different samples. PCA could reflect the differences and distances between samples by analyzing their feature compositions. The PCA results (Figure 5A) revealed that the bacterial communities of 4 °C-stored samples were clustered, and explained 89.21% (PCA1) and 4.22% (PCA2) of the variation. Fresh and pickled swimming crabs in different quadrants showed significant differences in bacterial communities. The same results were obtained for 4 °C-stored samples, which exhibited similar clustering (Figure 5B).

**Figure 6:**
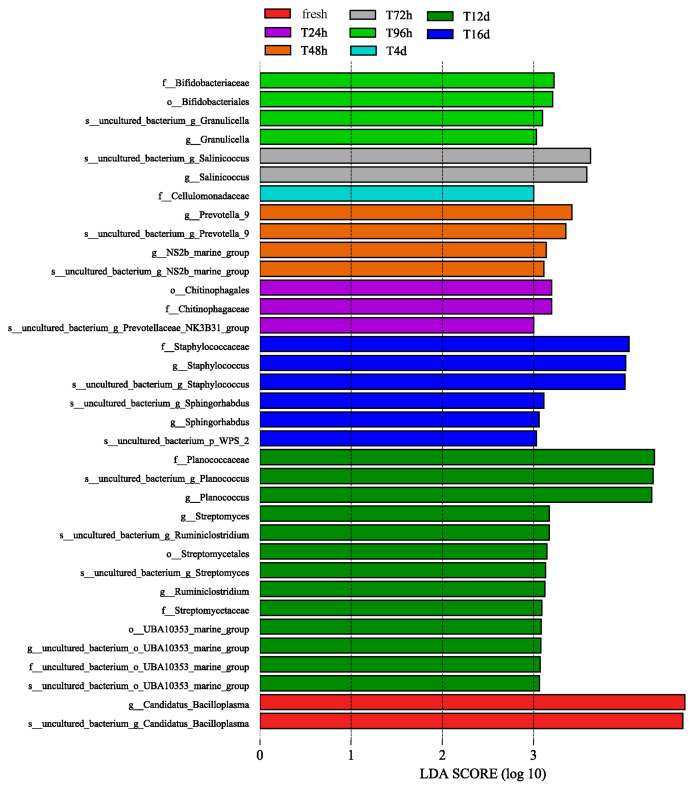
LDA value distribution histogram, which shows the significantly different bacteria with LDA value > 3.

To search for a specific bacterial genus of crabs during storage, we identified groups using an LDA effect size (LEfSe) analysis of significantly different species. The LDA values of the microbial groups with significant effects in different groups were calculated using the LDA analysis. Bacterial communities with significant differences were identified during sample storage using LEfSe analysis (Figure 6). The length of the bar chart represents the impact of different species, and the longer the length, the greater the contribution to intergroup differences. Excluding unclassified items, *f_Planococcaceae*, *f_Staphylococcaceae*, *g_Candidatus_Bacilloplasma*, *g_Planococcu*, and *g_Staphylococcus* were significantly higher in the fresh, 12 d, and 16 d groups.

## 3. Materials and Methods

### 3.1. Raw Materials and Pickled Swimming Crab Processing Procedure

Fresh swimming crabs (*Portunus trituberculatus*) weighing 212.36 ± 10.85 g were purchased from the local aquatic market (Donghe Market, Zhoushan, China) and immediately thoroughly scrubbed under running water. Two hundred fresh crabs were placed in a glass jar containing saturated salt water and alcohol (5%) at a volume ratio of 10:1 and refrigerated at 4 °C for 12 h. Subsequently, the pickled crabs were drained for 10 min and packed into polyethylene containers. One hundred pickled crabs were stored in a 20 °C incubator for 96 h, and the remaining one hundred were stored in a 4 °C of refrigerator for 16 d. The inedible parts were removed, and swimming crab abdominal muscles were obtained for various indicators tests. All chemicals used were of analytical grade.

### 3.2. Sensory Analysis

The sensorial acceptability of the samples was evaluated using quantitative descriptive analyses (QDA) with twenty volunteer trained panelists (ten men and ten women) [27]. All panelists were asked to assign acceptance scores for the appearance, tissue structure, flavor, and taste using a three-point hedonic scale. Score 0 represented the best quality, score 1 represented the endpoint of high-quality life (HQL), and score 2 represented the endpoint of shelf life (SL). Herein, we must address that the storage temperatures were different; we could not analyze quality of crab samples at the same storage time. Thus, the sensory evaluation was performed every 12 h for crab samples stored at 20 °C, and every 2 days for samples stored at 4 °C, respectively. The average score of each attribute was calculated.

### 3.3. Total Volatile Base Nitrogen (TVB-N) Determination

TVB-N value was determined using a steam-distillation procedure, according to Parlapani et al. [28]. Two grams of minced pickled crab meat were homogenized in 8 mL 6% trichloracetic acid. The sample was centrifuged at 1000 rpm for 10 min. Then, the supernatant was filtered using Whatman No.1 filter paper for analysis. TVB-N was released with the addition of saturated K_2_CO_3_, and was absorbed by a boric acid solution. Titration was carried out with 0.02 N HCl. The TVB-N content is expressed as mg N/100 g ± standard deviation of three replicates.

### 3.4. Microbiological Analysis

Microbiological analysis was carried out, according to Lorentzen et al. [5]. The pickled crab sample was homogenized with 45 mL of 0.85% NaCl solution in a filter stomacher bag using a Stomacher^®^ 400 Circulator (Seward Limited, Worthing, UK) for 1 min, followed by 10-fold dilutions using sterile 0.85% NaCl solution. Next, 0.1 mL of the mixture was spread on aerobic plate count agar to enumerate TVC. The plates were incubated at 30 °C for 72 h, then bacterial colonies were counted. TVC values were reported as logarithmic average values of the parallels and expressed as log (cfu/g).

### 3.5. pH Analysis

The crab meat was homogenized with 100 mL deionized water then filtered using Whatman No. 1 filter paper. The pH of the mixture solution was measured using a pH meter (METTLER TOLEDO, Shanghai, China).

### 3.6. Biogenic Amines (BAs) Analysis

BAs of samples were performed, as described by Qian et al. [29]. Briefly, 2 g of whole crab body meat were homogenized with 10 mL perchloric acid (HClO_4_, 0.4 mol/L) in a 50 mL polypropylene conical tube, followed by centrifuging for 10 min at 3000 rpm, after which the supernatant was filtered through a 0.45 μm pore Millipore filter. The precipitate was repeatedly washed with 10 mL HClO_4_ and centrifuged as above. Then, 100 μL NaOH (2 mol/L), 300 μL saturated NaHCO_3_, 2 mL dansyl chloride solution (10 mg/mL acetone) were added to 1 mL of the prepared filtrate solution. The mixture was incubated in a water bath at 40 °C for 45 min in the dark. After this reaction, 100 μL ammonia (25%) was added to the solution, which was stored at room temperature (25 ± 0.5 °C) for 30 min. Then, dansylated extract solution was adjusted to 5 mL using 0.1 mol/L ammonium acetate to acetonitrile at a 1:1 ratio, followed by filtering through a 0.45 μL pore Millipore filter. Ten microliters of solution were injected for HPLC analysis. The HPLC was equipped with a diode array detector (LC-10Avp series; Shimadzu, Kyoto, Japan) and C18 column (4.6 × 150 mm, shim-pack, Shimadzu, Kyoto, Japan). A mixture of methanol and water (V:V 70:30) was used as the isocratic mobile phase, with a flow rate of 1 mL/min at room temperature. The dansyl derivatives of the BAs were quantified by measuring the UV-absorption at 254 nm.

### 3.7. DNA Extraction and High-Throughput Sequencing

The pickled crabs were placed on an anatomical disk, the body surface was disinfected with alcohol, the abdominal muscle was removed in a sterile environment, and the sample was placed in a sterile centrifuge tube. After homogenization of muscle samples, DNA was extracted using a TGuide S96 Magnetic DNA Kit (Tiangen Biotech (Beijing) Co., Ltd., Beijing, China) according to the manufacturer’s instructions. The 338F: 5′-ACTCCTACGGGAGGCAGCA-3′ and 806R: 5′-GGACTACHVGGGTWTCTAAT-3′ universal primer set was used to amplify the V3-V4 region of 16s RNA gene from the extracted genomic DNA. Both forward and reverse 16s primers were tailored to sample-specific Illumina index sequences for deep sequencing. For the constructed library, Illumina NovaSeq 6000 (Illumina, San Diego, CA, USA) was used for sequencing analysis. All samples were analyzed by the Biomarker Biological Testing Company. The data were analyzed using the BMK Cloud [30]. The bacterial communities of fresh crabs were also measured as controls. Each sample was tested in triplicate.

### 3.8. Statistical Analysis

All experiments were replicated in triplicate (n = 3) for each group, and the average values are expressed as the mean ± standard deviation. Duncan’s analysis of variance (ANOVA) was performed using SPSS statistical software (SPSS Inc., Chicago, IL, USA; Version 21.0), and statistical significance was set at *p* < 0.05. Operational taxonomic units (OTUs) were obtained using an Illumina NovaSeq 6000 platform (Illumina, Santiago CA, USA) for high-throughput sequencing data. The heatmap of species abundance was plotted using the R project pheatmap package (version 1.0.12). The Alpha diversity was evaluate by QIIME (version1.9.1) and Beta diversity was calculated by GuniFrac package (version 1.0) in R project. Furthermore, online analysis tools (http://huttenhower.sph.harvard.edu/lefse/, accessed on 11 September 2023) were used for LEfSe analysis, and a logarithmic LDA score of 3.0 was set as the threshold for discriminative features.

## 4. Conclusions

In this study, the sensory scores and biochemical properties of pickled swimming crabs were investigated to study how storage conditions influence their quality and safety. According to the sensory analysis, TVC, TVB-N, pH and BAs, the shelf life of pickled crabs was 36 h and 6 days for samples stored at 20 and 4 °C, respectively. BAs data revealed that tyr, put, and cad were the major amines present during storage. Compared with another three BAs, all samples had the lower try, spd and hist contents, and these three BAs only could be detected during the product’s shelf life. The high-throughput sequencing results showed that the microbial complexity gradually decreased during cold storage. The salt–alcohol water mixture could significantly inhibited the growth of *Tenericutes*; in contrast, *Firmicutes*, *Proteobacteria*, *Bacteroidetes*, *Acidobacteria*, *Actinobacteria* and *Cyanobacteria* were the dominant bacteria in the stored samples. Compared with 20 °C, storage at 4 °C significantly protected the quality and food safety of pickled swimming crabs by inhibiting the growth of dominant bacteria. Overall, the present study provides some basic yet valuable information on pickled swimming crabs, which could enhance customers’ understanding of instant food items.

## Figures and Tables

**Table 1:** Changes in bacterial proportion and composition at phylum level in differently stored pickled swimming crabs.

Samples		Firmicutes	Proteobacteria	Bacteroidetes	Tenericutes	Acidobacteria	Actinobacteria	Cyanobacteria	Chloroflexi	Verrucomicrobia	Fusobacteria	Others
Fresh		27.19%	26.63%	15.73%	13.71%	3.02%	2.86%	2.11%	1.66%	1.65%	1.21%	4.22%
4 °C	4 d	25.09%	44.82%	14.65%	0.41%	3.05%	2.91%	1.45%	1.48%	1.10%	1.10%	3.92%
	8 d	38.30%	25.26%	17.21%	0.46%	3.68%	3.09%	1.64%	1.83%	1.62%	1.81%	5.11%
	12 d	40.95%	28.04%	13.77%	0.33%	3.44%	3.47%	2.13%	1.32%	1.37%	1.53%	3.66%
	16 d	34.66%	29.31%	16.54%	0.44%	3.83%	3.35%	1.85%	1.89%	1.44%	1.35%	5.34%
20 °C	24 h	34.39%	25.47%	18.87%	0.56%	4.01%	3.46%	2.12%	2.06%	1.71%	1.60%	5.75%
	48 h	37.50%	26.63%	16.07%	0.46%	4.21%	3.78%	2.33%	1.41%	1.64%	1.54%	4.43%
	72 h	38.80%	24.72%	16.81%	0.47%	3.98%	3.20%	1.74%	1.88%	1.59%	1.55%	5.27%
	96 h	35.43%	25.10%	17.61%	0.61%	4.29%	3.89%	2.28%	1.97%	1.65%	1.60%	5.54%

**Table 2:** Richness and diversity indexes of differently stored pickled swimming crabs.

Samples		Coverage	Alpha Diversity			
			ACE	Chao1	Simpson	Shannon
	Fresh	0.9973	2282.4731	2285.9071	0.9604	8.5563
	4 d	0.9980	2259.5870	2275.9723	0.9953	9.5851
4 °C	8 d	0.9980	2256.2301	2267.0133	0.9938	9.4586
	12 d	0.9975	2252.7042	2259.9469	0.9924	9.1744
	16 d	0.9980	2265.2872	2276.1444	0.9895	9.2712
	24 h	0.9982	2267.7451	2278.8716	0.9960	9.6734
20 °C	48 h	0.9979	2258.9893	2263.6768	0.9938	9.3706
	72 h	0.9981	2267.6745	2280.7749	0.9942	9.4571
	96 h	0.9979	2264.1723	2281.5283	0.9955	9.6389

Three parallel samples (n = 3) were set in each group. Values are the means from three samplings.

## Data Availability

The data presented in this article are available on reasonable request from the corresponding author.
